# Barriers and facilitators of tuberculosis infection prevention and control in low- and middle-income countries from the perspective of healthcare workers: A systematic review

**DOI:** 10.1371/journal.pone.0241039

**Published:** 2020-10-21

**Authors:** Charlene Tan, Idriss I. Kallon, Christopher J. Colvin, Alison D. Grant

**Affiliations:** 1 TB Centre, London School of Hygiene & Tropical Medicine, London, United Kingdom; 2 Division of Social and Behavioural Sciences, School of Public Health and Family Medicine, University of Cape Town, Cape Town, South Africa; 3 Department of Public Health Sciences, University of Virginia, Charlottesville, Virginia, United States of America; 4 Department of Epidemiology, Brown University, Providence, Rhode Island, United States of America; 5 School of Laboratory Medicine and Medical Sciences, College of Health Sciences, University of KwaZulu-Natal, Africa Health Research Institute, Durban, South Africa; 6 School of Public Health, University of the Witwatersrand, Johannesburg, South Africa; The University of Georgia, UNITED STATES

## Abstract

Tuberculosis remains a leading cause of death worldwide. Transmission is the dominant mechanism sustaining the multidrug-resistant tuberculosis epidemic. Tuberculosis infection prevention and control (TBIPC) guidelines for healthcare facilities are poorly implemented. This systematic review aimed to explore the barriers and facilitators of implementation of TBIPC guidelines in low- and middle-income countries from the perspective of healthcare workers. Two separate reviewers carried out an electronic database search to select qualitative and quantitative studies exploring healthcare workers attitudes towards TBIPC. Eligible studies underwent thematic synthesis. Derived themes were further organised into a macro-, meso- and micro-level framework, which allows us to analyse barriers at different levels of the healthcare system. We found that most studies focused on assessing implementation within facilities in accordance with the hierarchy of TBIPC measures—administrative, environmental and respiratory protection controls. TBIPC implementation was over-estimated by self-report compared with what researchers observed within facilities, indicating a knowledge-action gap. Macro-level barriers included the lack of coordination of integrated HIV/tuberculosis care, in the context of an expanding antiretroviral therapy programme and hence increasing opportunity for nosocomial acquisition of tuberculosis; a lack of funding; and ineffective occupational health policies, such as poor systems for screening for tuberculosis amongst healthcare workers. Meso-level barriers included little staff training to implement programmes, and managers not understanding policy sufficiently to translate it into an IPC programme. Most studies reported micro-level barriers including the impact of stigma, work culture, lack of perception of risk, poor supply and use of respirators and difficulty sensitising patients to the need for IPC. Existing literature on healthcare workers’ attitudes to TBIPC focusses on collecting data about poor implementation at facility level. In order to bridge the knowledge-action gap, we need to understand how best to implement policy, taking account of the context.

## Introduction

Tuberculosis (TB) remains one of the top 10 causes of death in the world, causing an estimated 1.45 million deaths in 2018, of which 250,000 were among HIV-positive people [1]. Additionally, there is increasing concern regarding multidrug resistant (MDR) and rifampicin-resistant (RR) TB. Their incidence have increased, with 3.5% of first TB episodes being MDR/RR-TB in 2017 [2]. It is now recognized that primary transmission is the dominant mechanism sustaining the global epidemic of drug-resistant (DR) TB [3, 4].

Nosocomial transmission of TB and DRTB remains an important mode of transmission. The 2005 outbreak of extensively drug-resistant TB at the Church of Scotland Hospital in KwaZulu-Natal, Tugela Ferry, highlights the risk of nosocomial infection towards patients [5]. This was particularly serious in a setting providing care to HIV-positive patients [5]. This risk extends to healthcare workers [6, 7], who are a critical asset in areas with a high burden of TB where healthcare systems are often overstretched and understaffed. The 2019 Global Tuberculosis Report published by the WHO found the ratios of the TB notification rate among healthcare workers to be higher than that of the general adult population in many of the high TB burden countries [1].

Despite the presence of TBIPC guidelines in most facilities and at a national level, implementation is usually limited. We undertook a systematic literature review with the aim of identifying barriers to and facilitators of TBIPC implementation in low and middle-income countries (LMIC) from the perspective of healthcare workers.

## Methods

The authors received no specific funding for this work.

### Eligibility criteria

We Included only studies published in English, with no restriction on the earliest date of publication up to 12 May 2020, which explored barriers or facilitators of TBIPC implementation by healthcare workers. We restricted the review to studies conducted in LMIC, because the prevalence of TB is higher, thus healthcare workers in LMIC have an increased risk of nosocomial transmission [8]. We included both medical and non-medical healthcare workers in all levels of healthcare facilities. Both quantitative and qualitative studies were included with no limitations on study design. Studies with multiple methods of data collection were included, on the basis that different research methods could offer different perspectives.

Studies which merely surveyed TBIPC implementation rates without investigating explanatory factors were excluded. Unpublished studies, opinion pieces and letters were also excluded.

### Search strategy

**Fig 1:**
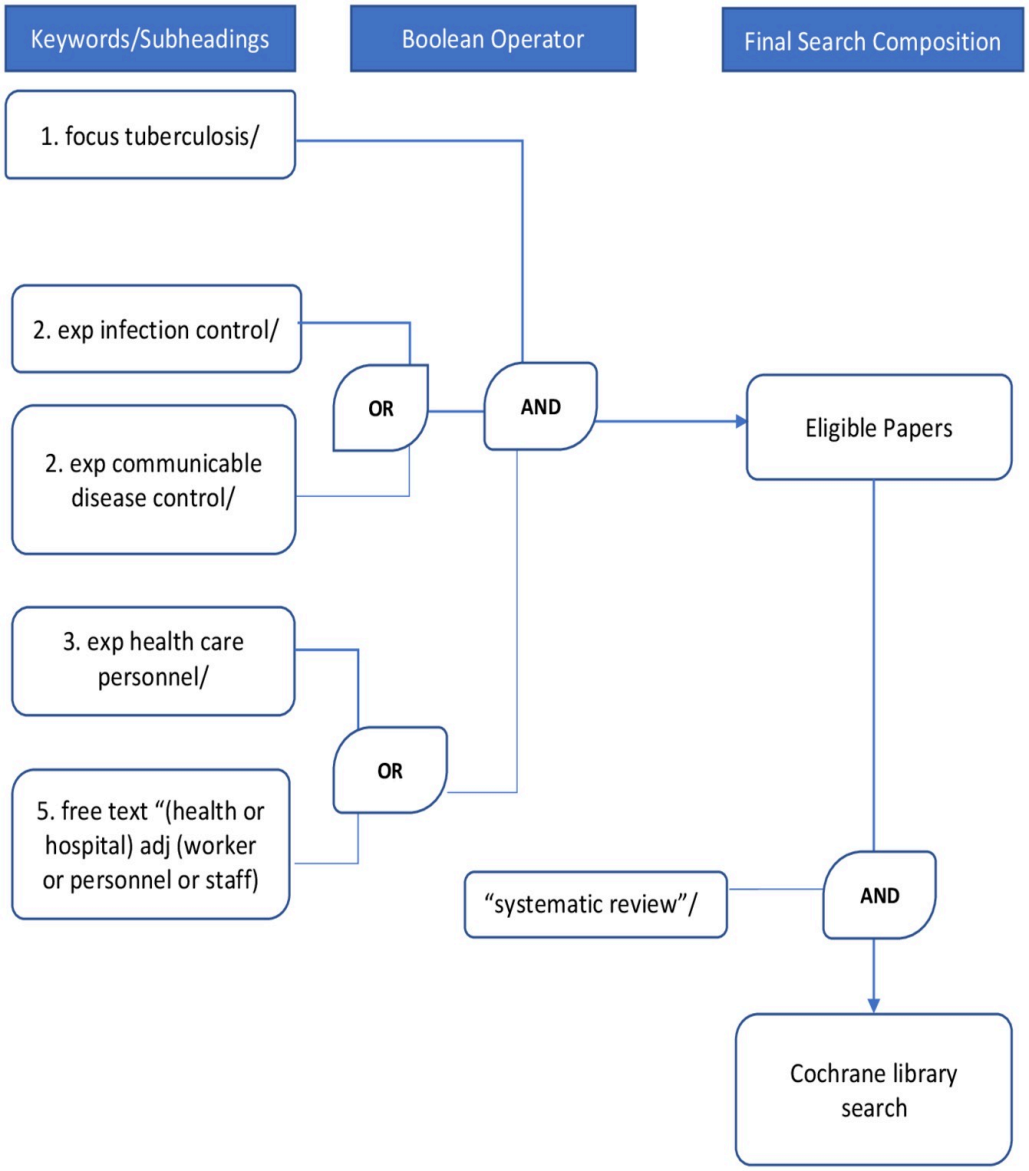
Literature search strategy.

The search strategy, which was developed with the help of a librarian, was refined in EMBASE using the OVID interface (Fig 1) and repeated in Global Health and Medline. The search was repeated in Cochrane to ensure there were no previous similar reviews and updated using Google Scholar. The search was constructed from controlled vocabulary (MeSH headings) and free text. The following concepts were developed in the search and combined using the Boolean operator “AND”

- Focused search on the MeSH heading “tuberculosis”
- Exploded MeSH headings “infection control” and “communicable disease control” were combined using Boolean operator “OR”
- Exploded MeSH heading “health care personnel” was combined using the Boolean operator “OR” with free texts “(health or hospital) adj (worker or personnel or staff)”.

Two reviewers (CT, IK) conducted the search independently, reviewing the titles and discarding papers with clearly irrelevant titles, and going through the abstracts of papers with possibly relevant titles. We then compared the relevant articles we each found in the three databases and resolved any disagreements by revisiting the aim to determine if the paper could contribute to answering the question. The reference lists of all relevant papers were manually scanned for further additional articles that met our criteria.

### Data extraction

Data extraction was guided by the Cochrane Data Extraction Template. Extracted information included that of study designs and methods, type of healthcare facility, type of healthcare worker, objectives and aims of study, information relating to factors which influence TBIPC practice, suggested measures to improve TBIPC implementation, and themes generated by both quantitative and qualitative studies. Data extraction was carried out independently by two separate reviewers and summarized in S1 Table.

### Critical appraisal methods

We assessed quality using the Consolidated Criteria for Reporting Qualitative Research (COREQ) [9] for qualitative studies and The Quality Assessment Tool for Quantitative Studies designed by the Effective Public Health Practice Project (EPHPP) [10] for quantitative studies. The “Blinding” and “Interventional Integrity” component ratings were removed from the EPHPP tool as they were not applicable to the quantitative studies. Hence the studies were graded based on selection bias, confounders, data collection methods and analysis. For studies using mixed methods, the quality assessment tool used was determined by the main method of data collection. For example, two knowledge, attitude and practice surveys also conducted an observation of TBIPC practice, aimed at validating the self-reported cross-sectional data, hence these were assessed as quantitative cross-sectional studies.

### Data analysis, synthesis and organization

Thematic synthesis was used for data analysis. The reviewers familiarized themselves with the extracted data on the barriers and facilitators of TBIPC. The data was organised using the macro-, meso- and micro-framework, as suggested by reviewer AG. This is a variation of Goffman’s frame analysis, which was used to allow the identification of barriers within and between the different stages of the transformation of policy to practice. This framework has been used in bridging the gap from translating research-based policy into application at a facility level within a healthcare system [11]. The macro-level frame examines barriers at a government level, such as policy and funding; the meso-level framework focuses on the programme (at a district level) which has been developed to implement the policy; the micro-level framework is concerned with the intricacies of implementing the programme at an organizational level (within the facility) in its day-to day function.

Within each level of the framework, two reviewers (CT) then identified broad themes. The reviewers then revisited the extracted data to generate key themes from the broader themes within each level (Table 1). The themes were finalised by all four reviewers during team discussions. Each theme was then written up, with the inclusion of quotes from the qualitative studies, and comparing similarities in findings amongst the papers whilst also recognising contrasting evidence.

**Table 1:** Synthesis of key themes.

Framework	Broad themes	Key Themes
Macro	Concern regarding the TB/HIV co-epidemic (in countries and facilities where this is applicable) Occupational health not prioritised/poorly implemented Difficulty incorporating occupational health into IPC No guaranteed reassignment of staff with HIV No ‘safer’ assignment (in TB specialist facilities) Funding prioritised towards other areas of healthcare Poor infrastructure—no isolation facilities Equipment not maintained Shortage of human resources	Lack of consideration of IPC in HIV and TB integration Ineffective occupational health policies as part of TB IPC Shortages of funding and resources
Meso	No guidelines within individual organisations Contradicting local and national guidelines Guidelines inapplicable at facility level No healthcare worker involvement No Staff training Lack of knowledge on TBIPC Selective training of staff	Transition from policy to programme Staff training
Micro	Non-approved respirators Inconsistent use of N95 Poor leadership at facility level Poor dissemination of guidelines to staff Poor working relationship between healthcare workers and TBIPC managers Poor practice passed on from old to new staff Feeling powerlessness Low morale from longstanding poor practice No danger pay Feeling undervalued Blame culture when TB contracted Patients non-compliant with IPC Communication barrier between patient and healthcare worker (in communities with different cultures or languages) Stigma surrounding TB Stigma surrounding HIV Stigma of masks Maintaining confidentiality after screening Concern for their own family Duty of care towards patients Importance of empathy and patient rapport Desensitisation to own risk Resigned to acquiring nosocomial TB Risk-benefit ratio Under reporting causes lack of awareness Impact of witnessing colleagues contract TB	Shortage of respirators Lack of authority to implement TBIPC programme Work Culture Difficulty in educating and sensitizing patients Stigma Healthcare workers perceived risks towards others Healthcare workers perceived risks towards themselves

## Results

**Fig 2:**
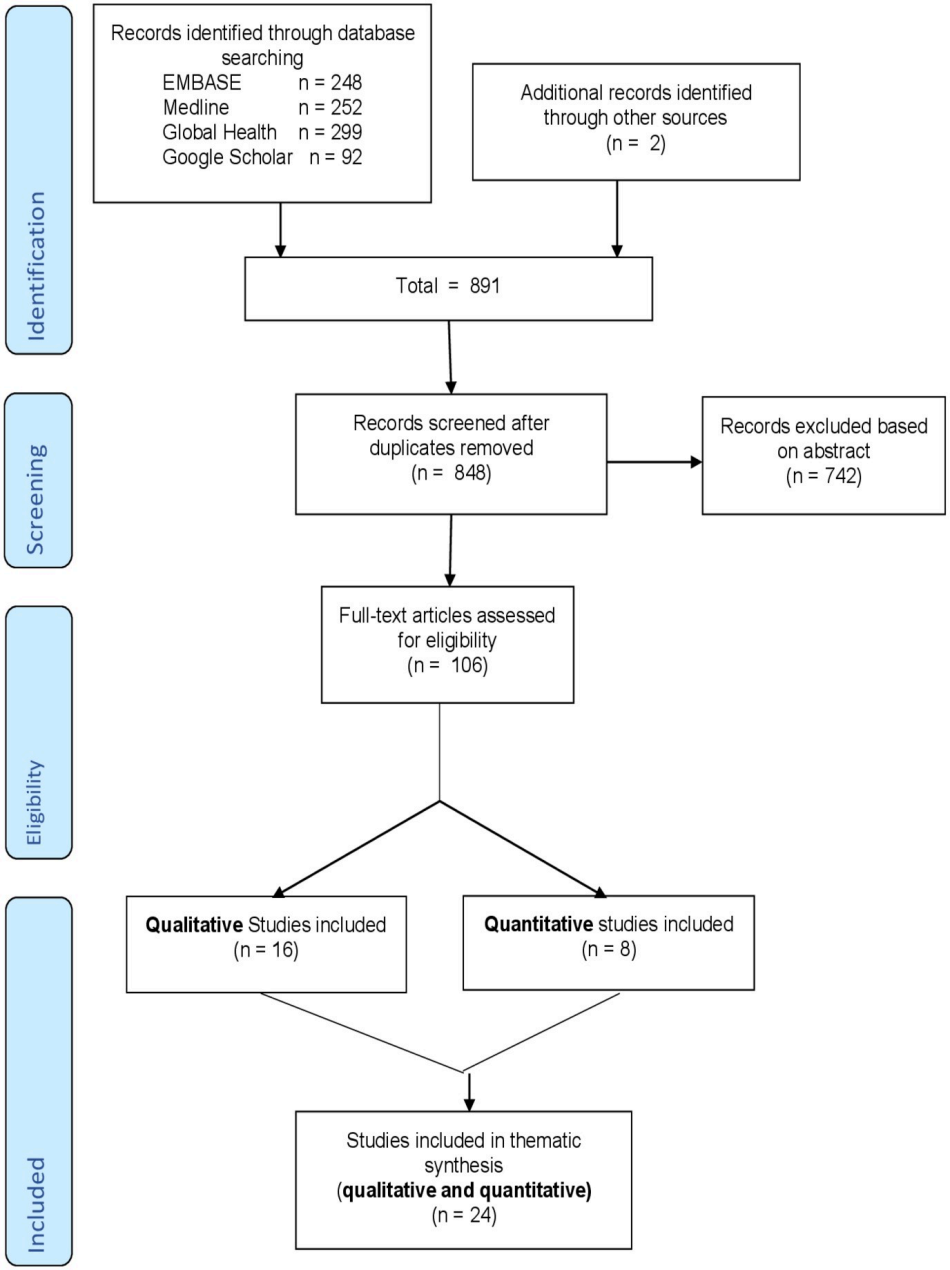
PRISMA flow diagram.

The search yielded 248 papers from EMBASE, 252 from Medline, 299 from Global Health and 92 papers from Google Scholar. This totaled 891 studies (Fig 2). After removing duplicates and reviewing the remaining abstracts, 22 eligible studies were identified and a further two were identified by manually checking reference lists. The results were then compared and 15 distinct disagreements resolved by consensus; the data were then compiled into a single table (S1 Table). In total 16 qualitative studies and eight quantitative studies were included (Fig 2).

### Characteristics of eligible studies

The twenty-four eligible studies were published between 2011 and 2020, originating from twelve countries, including South Africa (eleven studies), Ethiopia (two studies) and the Dominican Republic (two studies). Discussion sections of quantitative studies identified barriers that were similar to themes in qualitative studies.

Focus group discussions (FGDs) were the most common method used (nine studies). Four qualitative studies used in-depth individual interviews, two intervention studies conducted a follow-up group feedback session with participants and collected data informally via these sessions, and five studies included the observation of the participants’ TBIPC practice (not as the main method), carried out to validate self-reported data obtained from questionnaires. Five of the eight quantitative studies were knowledge, attitude and practice studies.

Only three of the studies assessed interventions. One conducted in South Africa, involving healthcare worker trainees, was a knowledge, attitude and practice questionnaire done before and after an TBIPC education programme based on the health belief model [12]. The second study conducted in Romania implemented a framework at TB facilities across the country, aimed at assisting these facilities in tailoring their TBIPC programmes to be site-specific [13]. Feedback was then gathered from participants on the barriers they faced during implementation. The final interventional study was a four-day TBIPC training course conducted at a training centre in Tajikistan. This included learning to plan and design TBIPC activities in accordance with national and international IPC guidelines and to conduct monitoring and evaluation activities for these programmes. A feedback session was conducted ten months later to evaluate participants’ retention of TBIPC knowledge over time, and to determine if participants had been able to implement any changes to TBIPC practices at their work places, using the skills they learned [14].

Found (two in South Africa [15, 16], one in Uganda [17], one in Nigeria [18]) of the five studies found that self-reporting overestimated the implementation of TBIPC practice when compared with the actual adherence rates observed by the researchers. The last study from Ethiopia did not include the results of the researcher-observed practices [19].

### Quality of studies

Five of eight quantitative studies were knowledge, attitude and practice studies [12, 15, 16, 20, 21]. As the questions and scoring systems were constructed by individual research teams and not validated, it was not possible to derive a meaningful comparison of the knowledge, attitude or practice scores (S2 Table).

Amongst the qualitative studies, methods for data analysis were similar across the studies with the main limitations being inadequate reporting on domain 1 of the COREQ checklist (Table 2). This domain pertains to the participation and role of researchers, and the importance of reflexivity. The majority of the studies had adequate reporting of domains 2 and 3, which are the study design, and the study analysis and findings respectively. Reflexivity was only considered by the researcher in one study [22], and only one study conducted in South Africa [23] made use of the COREQ checklist to ensure complete and transparent reporting.

**Table 2:** Quality assessment tool for qualitative studies using the COREQ (COnsolidated criteria for REporting Qualitative research) checklist.

	Adu 2020	Brouwer 2014	Buregyega 2013	Chapman 2017 ^(1)^	Chapman 2017 ^(2)^	Emerson 2016*	Kuniyu 2019^#^	Scott 2017*	Sissolak 2011	Tamir 2016^#^	Tshitangano 2015	Tudor 2013^#^	Turusbekova 2016*	Woith 2012	Zelnick 2013	Zinatsa 2018
**Domain 1: Research team and reflexivity (score out of 8)**	6	5	4	4	4	1	0	2	2	0	1	0	1	2	0	3
**Domain 2: Study design (score out of 15)**	13	10	10	10	11	2	9	6	10	4	10	4	3	9	6	10
**Domain 3: analysis and findings (score out of 9)**	7	7	5	8	8	1	5	5	6	6	6	3	2	6	4	7
**Total Score (out of 32)**	26	22	19	22	23	4	14	13	18	10	17	7	6	17	10	20

^(1)^ Perceived Barriers to Adherence to Tuberculosis Infection Control Measures among Health Care Workers in the Dominican Republic. MEDICC Rev. 2017;19(1):16–22.

^(2)^ The Role of Powerlessness Among Health Care Workers in Tuberculosis Infection Control. Qual Health Res. 2017;27(14):2116–27.

* Interventional Studies

^#^ Mixed-method studies—Including cross-sectional data, hence there was slightly more limited reporting on the qualitative methods.

The studies involving interventions aimed at improving TBIPC, such as the studies from Tajikistan [14], Romania [13] and the study conducted in both Zambia and Botswana [24] did particularly poorly in all domains when assessed with the COREQ checklist. However, this is understandable as their aims were focused on evaluating the effectiveness of their interventions. We chose to include them in this review due to the additional insight they provided on barriers to TBIPC. Similarly, three of the studies which used mixed methods [18, 19, 25], were found to have less adequate reporting on their methods, likely due to the word count being shared between multiple methods. One particular study from South Africa [26] scored poorly on the COREQ checklist relative to other studies. However, their results were in line with that of other studies and hence did not change the overall findings. There were no studies that were of sufficiently poor quality to warrant exclusion from this review.

### Combined qualitative and quantitative data analysis

#### Macro-level barriers and facilitators

*Lack of consideration of IPC in HIV and TB integration*. The study from Uganda conducted in 51 health care facilities noted that the work flow of their hospital neglected to take into account the need to separate TB patients from HIV patients [17]:

*“…the worst thing is that these HIV patients are seated together with TB patients and some of them are suspects and not yet on treatment*.”

*Ineffective occupational health policies as part of TBIPC*. Poor screening of healthcare workers for TB and HIV was reported in the studies from South Africa and the Dominican Republic [8, 27]. The nationwide study from Romania reported an underdeveloped system for reporting TB amongst healthcare workers [13]. None of the hospitals had developed a way to discreetly reassign immunocompromised healthcare workers to lower risk environments as this risked exposing their HIV status. In South Africa, this led to healthcare workers avoiding TB screening [22, 26]. In TB specialist hospitals, staff felt there was no point in screening as there were no safer designations available within the facility [26]. One study in KwaZulu-Natal, South Africa, conducted across three district hospitals purposefully selected because they had MDR-TB wards, found that occupational health nurses were unable to guarantee reassignment for healthcare workers with HIV. The hospital had made employees sign forms declaring that they understood the risks and chose to work in a high-risk environment [25]. This study also found that occupational health nurses lacked training specific to their role relative to IPC nurses [25]. Using open-ended structured questionnaires, this study found that the occupational health nurses felt they did not have the authority to implement changes, whilst IPC nurses felt they had the authority to implement changes that they thought appropriate to protect the healthcare workers. Despite recognising the value of integrating TBIPC and occupational health, this was not carried out as they were often run by separate departments [23]. Occupational health nurses also reported that the lack of a health and safety officer was a barrier to investigating safety issues. A government official from South Africa explained that due to limited resources, the health of patients was prioritized over that of healthcare workers [23]:

*“*…*because there are not enough resources*, *they would rather channel resources to serving the public than those people assisting the public”*

*Shortages of funding and resources*. Poor funding and insufficient resources were the theme by far most frequently mentioned across all studies. Healthcare workers cited poor infrastructure, such as a lack of isolation space [17,24], poor clinic layout [28], windows opening in the wrong direction [29], and being unable to maintain equipment such as extractor fans as barriers to implementing isolation or environmental measures [17, 19, 26, 27, 30]. Because of insufficient funding to hire more staff, overstretched healthcare workers viewed cough screening, isolation and patient education as additional, non-essential tasks [14, 17, 19, 22, 30, 31]. Some interviewees in a Nigerian study felt that this was compounded by the fact that TB services in their hospitals were free and hence did not generate revenue for the centre. As a result, managers would prioritise available funding to other areas of the hospital [18].

#### Meso-level barriers and facilitators

*Transition from policy to programme*. Some facility managers in South Africa found it difficult to understand the written policy and hence felt ill-equipped to train their staff or clarify misconceptions [8]. A study conducted in two rural district hospitals in South Africa reported an absence of any formal policy within the health care facility, despite a national policy being available [32]. One of the healthcare workers from a study conducted in South Africa cited a contradiction between the Ideal Clinic Initiative and the National Core Standard [8]:

*“…the Ideal Clinic says you must not have a separate TB room*, *that TB falls under chronic patients*. *The National Core Standard says you must have a TB cubicle…”* Although the Ideal Clinic Initiative is aimed at improving the standard of care in primary health care facilities in general, it includes standards concerning TBIPC which are not harmonized with the National Core Standard. A lack of healthcare worker involvement in governance was identified as a barrier in implementation of policies. Interviewees in a South African study cited the example that human resource departments were often tasked with implementing protective and supportive measures to prevent the acquisition of occupational TB [23]. However, they often lacked the necessary clinical understanding to implement these programmes:

“*There is no direct access to the decision makers around TB implementation policy in SA*.”

Both studies from Romania and Tajikistan trained TBIPC managers to adapt TBIPC programmes to suit their facilities [13, 14]. The study in Tajikistan conducted a feedback session to evaluate the utility of such training. They reported an increased uptake of administrative controls, such as an increase in the organization of TBIPC activities within their facilities (from 37% before training to 73% after training), staff and patient education, and policies changes within their facility. The study in Romania reported a 77.7% use of their TBIPC template by participants who were TBIPC coordinators from individual facilities or counties. However, it is worth noting that only 18 of the 42 participants responded to the questionnaire, placing the results at risk of a responder bias.

*Staff training*. Healthcare workers in the Dominican Republic supported the use of health education programmes to improve adherence to TBIPC measures [27]. Various barriers were brought up regarding training. Two studies from South Africa reported that healthcare facilities provided no training at all [8, 22]. A study conducted in Ethiopia across four hospitals, including 326 healthcare workers, found that only 18.8% of respondents were trained in TBIPC. Studies in Ethiopia and South Africa found selective training of staff who worked in TB-specific roles, such as facility managers [32], TB nurses or managers [19, 30], while the Dominican Republic study found that staff were prioritized for training based on their educational attainments [27]. Prioritizing staff for TBIPC training seemed to promote an attitude that TBIPC is solely the responsibility of staff who receive training [30]. In the study conducted in Georgia, only 30% of 240 respondents in the National TB programme were able to correctly identify high risk TB groups [21].

#### Micro-level barriers and facilitators

*Shortage of respirators*. The absence or shortage of N95 respirators was a commonly raised obstacle [8, 18, 19, 26, 30, 31]. This is especially detrimental to the prevention of nosocomial transmission among healthcare workers in low-resource settings where often, due to the failure of administrative and environmental protection measures, there is an over-reliance on the use of personal respiratory protection [26]. In Ethiopia, N95 respirators were reserved only for healthcare workers in contact with people with MDR-TB [19]. In Georgia, only 36% of healthcare workers from the National TB Programme reported frequent use of respirators when working with patients being investigated for or known to have active TB [21]. In Nigeria, 13.4% of DOTS centres within both primary and secondary health facilities reported to have N95 respirators available for staff use. However, on direct observation of hospital practices, the use of N95 respirators by staff was observed in less than 10% of centres.

A continued lack of respirators was found to lead to an indifference towards use, as healthcare workers in Mozambique reported growing accustomed to not wearing them [30]. Staff in the Dominican Republic felt undervalued, questioning why something as basic as respirators were not readily available [31]. The study conducted in Georgia through the National TB Programme found that the only reliable predictor of respirator use was their availability [21].

*Lack of authority to implement TBIPC programme*. Most guidelines recommend that a TBIPC manager is appointed within a facility to disseminate the healthcare facility’s policy and conduct surveillance on TBIPC implementation within the facility. There were facilities in South Africa and Ethiopia with absent [16, 19] plans and missing standard operating procedures [22]. In South Africa, some healthcare workers were unaware of their health care facility’s TBIPC policy and others were unfamiliar with its content [8, 33]. The document in Lesotho found that 22% of healthcare workers were unable to access a copy of the facility’s TBIPC plan, some reported a single copy kept in the office of the TBIPC manager [33]. In Nigeria, it was found that of 112 DOTS centres, only 21.4% had a TBIPC plan and only 58% had a designated TBIPC officer [18].

A study from South Africa suggested the TBIPC officer did not have the authority to implement the guidelines:

*“Nurses do not wear N95 masks because they say they are uncomfortable*. *So what is a hospital manager supposed to do*?*”*

[26]

This was also brought up as one of the broader challenges by facility level TBIPC managers in the Romanian study [13].

TBIPC administrators who visited wards and offered verbal encouragement to healthcare workers in Russia were viewed as supportive [34]. Similarly, visits from district officials in South Africa to reassure facility managers that their TBIPC programme was robust was a motivating factor for the staff and managers [8].

*Work culture*. The studies in Uganda, Russia and Mozambique found that poor adherence to TBIPC measures, if not rectified, becomes entrenched in the work culture [17, 30, 34].

*“…we have worked here for years treating TB patients and none of us has ever got TB*. *Why the fuss now*?*”*

[17] (Uganda)

Poor adherence was also found to be passed from a senior to junior level in a different study of healthcare worker students in Uganda [35]. 66% of health Science students in a South African study feared possible academic consequences if they went against their seniors by adhering to TBIPC measures [12].

A South African Study reported a sense of powerless to change the way things are done [26].

*“This is such longstanding problems in the primary healthcare that even if you tell me it will change [by] December I will think it’s a big joke*!*’*

[8]

In South Africa, feeling undervalued and unsupported were attributed to reasons such not receiving danger pay when working with DR-TB, being unable to claim compensation despite acquiring TB nosocomially [26], management failing to implement new safety regulations when colleagues contract TB and blaming the healthcare workers for negligence when they contract TB [8]. Healthcare workers in a study from the Dominican Republic felt that other health goals such as maternal and child health [27] were prioritised over TB, while healthcare workers in another study in South Africa felt that HIV was prioritized over TB [22].

*Difficulty in educating and sensitizing patients*. healthcare workers in Zambia and Botswana brought up patient cooperation as a barrier to implementing TBIPC [24]. A study in South Africa reported that patients would refuse surgical masks distributed by healthcare workers [8]. When nurses and patients originated from different backgrounds or cultures, they faced a communication barrier when attempting to educate patients on TBIPC [22].

*Stigma*. Out of concern for the impact a TB diagnosis may have on how the community perceives a patient, healthcare workers were found to selectively implement TBIPC measures. The study from Mozambique reported a regression in the practice of isolating TB patients.

*“…more recently this practice [isolation] was considered discriminatory*, *he or she is a patient like any other patient and then we started to mix them with the others”*

[30]

Another study in Uganda found that the reason healthcare workers did not screen for TB symptoms in the waiting area was because they felt that the questions were too sensitive and should be asked in private during the consultation. In patients where a diagnosis was suspected but not confirmed, healthcare workers were uncomfortable informing the patient [17].

In attempt to build a closer patient-healthcare provider relationship, healthcare workers in Mozambique were found to forego the use of respirators [30]. Studies from both Mozambique and the Dominican Republic reported that healthcare workers perceive masks as both a physical barrier, preventing effective provider-patient dialogue, and an emotional distancing mechanism, displaying a lack of empathy [30, 31].

The stigma surrounding TB and HIV also impacted the decisions healthcare workers made regarding their own health. Two studies from South Africa found that healthcare workers were uncomfortable with attending occupational health screening for TB or HIV for fear of their colleagues breaching confidentiality [16, 26], and of being redeployed to a low-risk working environment, inadvertently exposing their HIV status. By contrast, one study in Ethiopia reported 84.8% and 87.5% of healthcare workers were comfortable with requesting their HIV and TB status respectively [19].

*Healthcare workers perceived risks towards others*. healthcare workers in Russia and Mozambique were motivated to adhere to TBIPC measures to avoid putting their family members at risk of acquiring TB [30, 34], whilst healthcare workers from studies in South Africa and the Dominican Republic feared spreading TB within the facility to other patients [22, 27] and other hospital staff [27].

The two studies from the Dominican Republic found that a strong sense of duty of care felt by healthcare workers towards their patients manifested itself in two contrasting schools of thought. While some felt a moral obligation to adhere to TBIPC measures to protect their patients, whom they felt were powerless to protect themselves [27], others prioritised empathy and building patient rapport over wearing respirators. They justified their actions by taking other precautions, such as keeping a distance from patients when speaking to them [31].

*Healthcare workers perceived risks towards themselves*. A desensitization to the risks of acquiring nosocomial TB was common. The study from Russia found that healthcare workers perception of their risk seemed to diminish with time [34], while the two studies from the Dominican Republic found that healthcare workers developed of a sense of invincibility [27, 31]. Other participants from these studies believed that most healthcare workers had already contracted TB [31, 34] or, in the case of South Africa, MDR-TB [8] and exposure was inevitable, hence TBIPC would be futile. Some believed that divine intervention was protective [8, 31].

The study conducted in Mozambique cited that healthcare workers felt empowered by knowledge. They argued that knowledge allowed them to manage the risks by taking precautionary measures with TBIPC [30]. Similarly, one study argued that the under-reporting of TB cases would dangerously underestimate the extent of nosocomial transmission within a facility and hence reduce awareness amongst healthcare workers of their risk [23]. In three studies from South Africa, healthcare workers reported that witnessing fellow colleagues contract MDR- and extensively drug-resistant TB (XDR-TB) initially served as a motivating factor [8, 22, 32], but this was quickly attenuated when no changes were made to improve working conditions.

A study conducted in Georgia [21] based on the health belief model found that healthcare workers were more like to refuse treatment for latent TB infection if they worked in TB facilities. They rationalised that they would shortly reacquire latent TB infection on completion of treatment, and hence this short-lived benefit outweighed the inconvenience of being on treatment. The study also found that those who perceived latent TB infection to be more serious were more willing to receive treatment.

## Discussion

Across the included studies, the most consistent concern reported by healthcare workers was the lack of funding and resources for effective TBIPC. Given the higher rate of hospital-acquired TB among healthcare workers in LMICs compared with high-income countries [36], it might be tempting to conclude that the key to successful TBIPC is simply increasing the resources available. However, there are low-cost, effective TBIPC interventions available as well as a range of existing assets within LMIC settings that can be leveraged. For example, some of the healthcare workers showed initiative in overcoming infrastructural limitations by setting up isolation areas outdoors when indoor waiting areas were overcrowded [17]. Common barriers to TBIPC, such as difficulties in changing work culture or failure to maximize resources (e.g. proper use of available respirators), do not require—or even benefit from—additional funding to address. For example, the Martin Preuss Centre in Malawi is a purpose-built HIV/TB integrated services clinic designed to facilitate infection control [37]. The design includes outdoor waiting areas and ART clinics which are situated on the other side of the building from TB clinics. Patients with and without TB are separated at the front desk by colour-coded signs. Although this low-cost solution to patient flow and segregation cannot be immediately replicated in existing facilities, and did require funding and collaboration between two separate agencies, it combines a simple layout with a well-thought out clinic flow, both of which do not require costly equipment or maintenance.

Training was another widely-covered theme. In line with a review on the role of education in IPC [38], we found that while training may improve knowledge, it does not always effectively or sustainably change practice. One of the main findings in this review was that there was a lack of robust evidence suggesting that education had a long-term effect on improving practice and reducing infection rates. One explanation could be a gap between knowledge and application, where the training focus is on theoretical knowledge rather than practical aspects of TBIPC [14]. Another reason could be a different kind of knowledge-action gap, widely observed in healthcare [39], where people know what to do and how to do it, but fail to change their practice nonetheless. This gap could be seen in the disparity between the observed TBIPC adherence by researchers and self-reported TBIPC adherence in questionnaires. This suggests that an awareness of the appropriate measures to be taken exists, yet there is a failure to carry them out. Whether it is a conscious or unconscious decision to omit appropriate practice is difficult to distinguish, Although the latter reason would be better described as a perception-action gap, the likely explanation is a combination of both.

Many of the micro-level barriers identified are resonant with social cognitive models, which have been used to explain the knowledge-action gap. For example, the Health Belief Model [40] suggests that healthcare workers’ perceived susceptibility to, and severity of, a disease has an impact on their behaviour. Those who perceived latent TB infection to be more serious were more likely to agree to treatment [21]. Our finding that work culture was an important influence on behaviour is in line with the theory of reasoned action, which argues that “normative beliefs”—an individual’s beliefs about what others expect and to what degree they wish to comply with these expectations—influence their decisions and behaviour. This would be in keeping with a conscious decision to carry out or omit the implementation of TBIPC. Alternatively it is argued that individuals are processors of routine tasks and find it challenging to integrate new processes into their routine [41]. This is supported by our findings of a long-standing culture of poor TBIPC practice within organisations.

Whatever the role of individual-level factors, however, we should recognize that gaps in translating knowledge into action usually reflect systems-related and structural issues and not solely provider behaviour [41]. A review of health behaviour theories in the development of interventions argued that not only do these theories occasionally contradict each other, but also that their applicability to contexts outside of which they were developed remains unvalidated. Further, they do not address health systems and social-cultural aspects sufficiently, such as the culture of doctor training or the stigma attached to TB due to its perceived relation to HIV [42]. Although most National TBIPC policies are based closely on the WHO guidelines, each country has unique political and/or social contexts influencing the healthcare systems and culture. This needs to be taken into account when translating nationwide policies into local organizational programmes, as recognized by the nation-wide study conducted in Romania [13]. Failure to take context into account helps to explain prominent IPC failures like the hand hygiene campaigns in the United Kingdom, which attempted to replicate the success of those in Geneva, but failed to take into account the contribution of contextual factors, such as the availability of isolation facilities, the effectiveness of bed management or the authority of infection control teams to close wards in the case of outbreaks, and health workers’ general awareness and training in relation to infection control [43]. Evaluators of the UK campaign concluded that “a customized intervention from another country that fails to consider local organizational factors likely to influence implementation of the campaign is unlikely to be effective”. Current literature suggests that healthcare workers functioning at a micro-level are expected to apply policies which are meted out by those working at a macro-level, who often fail to take into account the context in which these healthcare workers operate.

We found that both the quality and the scope of studies meeting our inclusion criteria was limited. Most studies focused on documenting poor practice at the different TBIPC levels, namely administrative control measures, environmental control measures, and respiratory protection. Although three of the five knowledge, attitude and practice studies concluded that poor adherence was due to insufficient knowledge, these results were derived from questionnaires about self-reported behaviour, which may be influenced by social desirability bias. The focus on healthcare workers’ practice assumes that poor TBIPC implementation is due to nonadherence by healthcare workers, which as discussed earlier, apportions blame to healthcare workers without considering system deficiencies. Knowledge, attitude and practice surveys also have the disadvantage of being restricted to questions and responses preset by the researcher. Qualitative studies are needed to uncover themes beyond those already known to the researcher. Most qualitative studies reviewed could have included more consideration of reflexivity.

Existing research under-represents the experience of IPC implementation in primary health care facilities. Primary health care facilities are usually smaller and less well resourced, and would face a different set of challenges to tertiary facilities, but are very important with respect to TB IPC because of the large numbers of patients who attend, particularly the increasing numbers of HIV-positive people who are more susceptible to developing tuberculosis.

There was also a lack of studies at the meso- and macro-levels, with only three of the twenty-four studies having been conducted at a national level (from Romania, Tajikistan and the last in both Zambia and Botswana) and one study from South Africa focusing on macro barriers to implementation [23]. This either reflects a shortage of literature at a meso- and macro-level, or a limitation of our search strategy. Our search, focused around terms related to “healthcare workers”, may have yielded results that were inclined towards the micro-level. This may have resulted in a proportion of the micro-level factors being associated with occupational transmission despite the focus of the review being on the implementation of all levels of TBIPC. It may be beneficial to examine policy documents and national guidelines to look at the limitations considered when writing these guidelines. However, this was not within the scope of our review as our focus was on healthcare workers perspectives. Another limitation of this study is the restriction to studies reported in English.

## Conclusion

The small number of available studies exploring the research question indicates that work is needed to achieve a more nuanced understanding of why implementation of TBIPC remains limited in many settings. The focus of existing literature is on documenting poor practice at the various levels of TBIPC and framing the problem as one of poor adherence to guidelines by healthcare workers. Future research should take more account of the broader context in which policy is implemented, both within individual healthcare facilities and the communities which they serve, in order to find effective solutions to protect healthcare workers and their patients from nosocomial transmission.

## Supporting information

S1 Table Data extraction table. (DOCX)

S2 Table Quality assessment tool for quantitative studies by the Effective Public Health Practice Project (EPHPP). (DOCX)

S1 Checklist PRISMA 2009 checklist. (DOC)
