# Supplementary material for: Barriers and facilitators of tuberculosis infection prevention and control in low- and middle-income countries from the perspective of healthcare workers: A systematic review
Source: PLoS One. 2020 Oct 21;15(10):e0241039. doi: 10.1371/journal.pone.0241039 (PMC7577501; doi:10.1371/journal.pone.0241039)
Supplement: S1 Table — (DOCX) [file pone.0241039.s001.docx]

**S1 Table. Data extraction table**

| **Author, Journal, Year, Country** | **Aims/objectives, Study type** | **Sampling/recruitment method, No of participants, Type of HCF, data collection method** | **Results** | **Reviewer Comments** |
| --- | --- | --- | --- | --- |
| Kuyinu  Int J Tuberc Lung Dis  2019  Nigeria, Largos State | 1. Assess the level of implementation of TBIPC measures 2. Assess air exchange rate in DOTS centres   **Qualitative** | 112 DOTS centres across all 20 local government areas   - Absolute number of participants not stated - Selection - Primary (both government and privately owned) and secondary health facilities - 5 FGDs with 8-10 members in each group, all from primary health facilities   Cross-sectional (for availability and implementation of TBIPC) + Direct Observation (of TBIPC practices) + FGD | Quantitative   - Managerial: 21.4% had TBIPC plan, 58% had designated TBIPC officer, 57% of staff received TBIPC training - Administrative: 67.9% screened for cough in waiting areas, 91% triage, 34% had non-touch waste-disposal bins, 63% have information on cough hygiene and etiquette for patients and staff - Environmental: 75% designated consulting rooms for TB activities, 6% had mechanical ventilation (ceiling or standing fans) - PPE: 13.4% reported to have N95 available for staff use (but only 10% on direct observation), 37% reported to provide facemasks for patients (only 29% on direct observation), poor ventilation if consultations held indoors   Qualitative   - Perceived barriers to TBIPC: centres not purpose-built, funding limitations, inadequate PPE and consumables, inadequate manpower, lack of training - Structural inadequacy: outdoor consultations which don’t allow maintenance of privacy or confidentiality, no designated area for sputum production - Availability of TBIPC measures: No formal training on TBIPC, No TBIPC committee - Implementation: No specific job role to triage coughing patients, N95 masks not used - Healthcare worker risk perception: (vaguely written) patients do not understand risk of transmission which affects how healthcare workers relate to them - Effect of risk perception on service delivery: causes stigma, workers assigned to DOTS placements refuse their posting, other healthcare worker avoid those who work with TB patients | - TBIPC plans in place but with poor implementation - No specific training for staff on TBIPC, no effort to train new staff - Gap in practice attributed to shortage of human resources - Management unwilling to prioritise TB services as they are free and not revenue generating - Stigmatisation worsens the shortage of human resources for TB - TBIPC measures overall better in secondary health care facilities compared to primary - Gap between self-reported and directly observed TBIPC practice - Poor political will - Poor implementation aggravates stigmatisation (unsure how they came to this conclusion) |
| Adu  Annals of Global Health  2020  South Africa | 1. To elicit the perceptions of informed person of the health system barriers to protecting the health of health workers 2. Examining how system factors are perceived by key actors in a high TB burden health system   **Qualitative** | 18 key informants recruited, purposive and snowball sampling (20 approached, two did not respond) between Oct and Dec 2016   - From Gauteng, Western Cape and KwaZulu-Natal - 10 government employees working in TB control - Four academic experts - Two TB advocacy group members - One legislator - One hospital leader   Semi-structured interviews | Focus on upstream or “macro” factors - political, economic, macro, organizational and cultural barriers   - Lack of priority afforded to protecting worker health: lack of effective leadership championing occupational health – unawareness amongst senior managers and political heads about occupational health. Channeling of scare resources to serving the public and not healthcare workers, inability to recognize the link between health worker retention and safe working conditions. A lack of accurate information about TB amongst healthcare workers as a specific barrier to systemic awareness and action - Governance and leadership: often taken up by human resources departments which lack knowledge or clinical understanding of healthcare worker health issues. Minimal involvement of healthcare workers who have no direct access to decision makers. One informant refuted this claim. Suggestion to integrate occupational health and IPC - Financing: insufficient allocation of resources, budgeting is a limiting factor - Health workforce for occupational health and IPC: shortage of well-trained staff, no effort to promote occupational medicine as a career path or create jobs in this area, no capacity building - Service delivery to health workers: confidentiality is an issue when getting tested for TB in the workplace - Protective technologies: poor environmental controls and protective equipment, difficulty maintaining and installing technology, little attention paid to non-clinical support staff | - Systemic barriers in particular include lack of priority afforded to occupational health and underestimation of risk to the health workforce - Underreporting causes a lack of awareness amongst managers and healthcare workers of the extent of increase in TB risk in the work place - Lack of funding despite high percentage of GDP spent on health due to HIV/TB co-epidemic - Leadership recognizes health workers as a key population for treating TB, but no concrete actions or funding has been allocated to address this - Despite the value in integrating IPC and occupational health, difficulty in doing this is recognized due to their originating from different components of the health service – IPC is seen as part of patient care and occupational health part of human resources - Need for a more active worker role in governance - Rather than employing qualified staff to run occupational health and IPC, managers tend to delegate the role to existing staff who are not trained in these areas. This is because they do not recognized IPC and occupational health as essential health system components. This makes them unattractive professional fields and exacerbates the skills shortage. - Stigma needs to be managed at a data security level, not just with education to address the attitudes and fears that cause stigma - Health systems inability to maintain/manage technology such as ultraviolet air disinfection |
| Zelnick  J Public health policy  2013 Aug  South Africa, KwaZulu-Natal | 1. Using frontline perspectives to identify challenges that healthcare workers face implementing measures to reduce TB risk 2. Expand their understanding of how to improve workplace safety   **Qualitative** | Recruited by hospital contacts during in-service training times and recruitment by researchers  5 hospitals in KwaZulu-Natal   - 61 healthcare workers: 55 from FGD + 7 from interviews - 1 TB referral hospital, 2 regional TB hospitals, 2 district hospitals - Rural and urban catchments   FGD + structured individual interviews | M: Lack of training. OH policies poorly implemented (linked to stigma): not reporting needle stick, not declaring HIV status  A: wait time for transfer to TB hospital, patient behavior/cough etiquette not addressed  E: Poor infrastructure - no windows, extractor fans broken, space for isolation  P: uncomfortable, unavailable in non-TB hospitals, unsure of use, lack of knowledge about effectiveness, contradictions between requirements and expense, staff were aware it was mandated  Others:   - Stigma around TB and HIV - No danger pay for DR-TB and impossible to get compensation for occupationally acquired TB - Perception that there is no ‘safer’ work environment in TB specialist hospitals - Hospitals don’t have authority to spend money to implement IPC policy - Healthcare workers suspected guidelines were influenced by economic concerns 🡪 doubting the safety of guidelines   Facilitators: annual TB screening – but only in TB hospitals, checking on compliance with mask use | 1. Lack of resources 🡪 unrealistic policies in view of gaps of resources and infrastructure. E.g.: 2. Delay in diagnostics 3. Lack of space 4. Shortage of beds 5. Distrust of IC efforts due to contradiction between policy and practice 6. Disproportionate focus on individual personal protection: E.g.: 7. Not always available 8. Not always fit testing done 9. Not always worn   Comprehensive IC policies but not implemented due to gaps in resources |
| Zinatsa  BMC Health Services Research  2018  South Africa | Using the IMB Model, identify factors that influence TB infection control practices and elicit recommendations to improve infection control in PHC facilities  **Qualitative** | Voluntary participation of staff who participated in a 2015 control survey  33 facility managers and 20 TB nurses  Primary health care facilities  5 FGDs | INFORMATION  Training: mostly for managers – in contrast to guidelines from National Department of Health  Policies: Too many policies, conflicting guidelines. Not tailored to PHC facilities  PPE: unsure of how to use, when to discard, how it protects.  MOTIVATION   - knowing a colleague who had fallen ill 🡪 mitigated by lack of changes in working conditions - Role-modeling by facility managers - Mentorship - Having an MDR patient - National Core Standard inspections - Workmen’s compensation   BARRIERS   - Low motivation - Powerlessness: PHC neglected - Poor attitudes - Stigmatization with masks - Poor support/Health system factors: staff shortage and poor infrastructure (singled out as having greatest impact), poor occupational health support | Suggestions of how to improve TBIPC   - Improving knowledge: Training to bridge the knowledge gap, refresher training, patient education and awareness, clarification of guidelines - Motivating: by emphasizing their role as agents for change, low cost rewards to acknowledge efforts - Strengthening behavioural skills: practical hands-on skills training/role-playing to increase self-efficacy, destigmatizing HIV and TB |
| Sissolak  BMC Public Health  2011  South Africa, Capetown | To explore factors influencing TB-IPC practices at a hospital level from the experiences of ward nurses in order to identify risks associated with potential nosocomial transmission, and to emphasize the essential role nurses play in TB control and care  **Qualitative** | Restricted sampling to those on off-duty plans whoever was available. No male nurses of African or white ethnicity. All participants were of coloured ethnicity.  20 nurses  Individual in-depth interview with semi structured interview guide | Barriers   - No designated TB wards - Lack of isolation facilities - Lack of knowledge of appropriate use of masks and respirators - No adequate TB training (Note, this resonates with Zinatsa reading published 7 years later and other readings below): continuous professional development only available to doctors and not nurses - No standard operating procedure: No precautions with suspected TB, results in inconsistencies in practice - No initiative, not empowered - Work overload - Communication barrier between staff and patients - More focus on HIV, TB neglected: more fear and stigma surrounding iatrogenic transmission of TB than HIV - Stigma in community   Motivators   - Strong sense of duty of care - Personal fears of contracting TB mitigated by misinformed health beliefs | - Healthcare system inadequacy was the major influence - Need for comprehensive TBIPC policy that reflects current guidelines, legislative and evidence of annual audits - Healthcare workers cannot be blamed for applying inconsistent TB-IPC measures when appropriate, accessible and clear guidelines are not implemented, monitored and evaluated - Shortage of staff 🡪 work overload 🡪 competing clinical priorities 🡪 high risk environment - Despite poor working conditions, there was a strong sense of a duty of care - deficit in knowledge of cross-cultural health beliefs |
| Tshitangano  Journal of Human Ecology  2015  Vhembe District, South Africa | To explore both employee and management factors that influence healthcare workers’ implementation of ineffective TB control measures in public rural hospitals  **Qualitative** | Seven of eight (one was psychiatric and did not take TB patients) rural hospitals in the district of Vhembe. Purposive sampling of one representative from each ward per study hospital, with the necessary knowledge.  57 Healthcare workers  Rural public hospitals  Focus group discussions   - 7 groups of 5- 10 members - pilot study at first hospital | Barriers   - Main theme: dynamics faced by Healthcare workers in rural hospitals - 7 subthemes:  1. Lack of clear directing TBIPC guidelines: inadequate infection control training with insufficient content – Empowerment model 2. Inappropriate designs of building: e.g. window that flip inwards, not permitting maximum air entry, or windows that open into other wards, rather than into the environment 3. Inadequate material resources: Examples centre around N95 masks 4. Inadequate human resources: infection control nurse not available in all hospitals 5. Inadequate management support: this was not well expanded upon with no data from FGDs themselves 6. Incorrect attitudes towards good TB control practices: poor attitude towards screening, excuses – difficult to stop patients walking around hospital 7. Inadequate knowledge regarding good TB practices: only mask usage was discussed | - Rural hospitals of Vhembe district do not have TB control plans - Training is required to empower Healthcare workers to implement TBIPC   (Mostly a reiteration of results and comparison to studies with similar findings. Not very developed) |
| Tudor  Public Health Action  2013  KwaZulu-Natal, South Africa | To increase understanding of the implementation of occupational health (OH) and infection control (IC) guidelines for the prevention and control of TB among healthcare workers  **Structured questionnaire with open ended questions** | Selected in collaboration with KwaZulu-Natal Provincial Department of Health (DOH)  6 participants = 3 OH and 3 IC nurses  Three District General Hospitals in KwaZulu-Natal with specialized MDR-TB wards  OH – 65-item structured questionnaire with open ended questions  IC – 21-item structured questionnaire with open ended questions  Chart review of OH employee medical records | - Hospitals under-reporting Healthcare workers TB cases - None of the three hospitals evaluated had a health and safety officer at the time of this study, and only one had an active health and safety committee - Barriers to implementing or enforcing OH guidelines  1. OH staff feel they have no authority to implement change 2. Lack of OH training 3. Absence of health and safety officer poses difficulty in investigating safety issues 4. Overworked and understaffed  - Barriers to re-assigning staff:  1. no guarantee of reassignment – one hospital made staff sign forms to say they understand the risks and choose to work in a high-risk environment 2. Process has to be two way – OH can ask about immunocompromised but it is up to staff to reveal their status or accept testing. Reassignment reveals HIV status and because of stigma, staff are reluctant to request for reassignment or get tested  - Non-approved respirators sold as N95 masks (this seems like a managerial lapse rather than available resources)   IC nurses feel more empowered than OH nurses | - Significant gaps between delivery of services and adherence to available recommendations and guidelines - Availability of employee medical records, TB screening and acceptance of HIV counselling and testing varied across sites, highest at the site that had a trained, full-time OH nurse, suggesting that OH training may lead to greater adherence to screening and prevention recommendations. - Majority of TB identified amongst staff when they were symptomatic, suggesting importance of routine screening - Integration of OH and IC services would be ideal. |
| Tamir  BMC Health Services Research  2016  Northwest Ethiopia, West Gojjam zone | To determine the level of practice on TBIPC and its associated factors among Healthcare workers  **Qualitative** + **Cross-sectional + Practice observation** | 35 (10 urban, 25 rural) Healthcare facilities selected from 91 centres using simple random sampling.  Questionnaire: Systematic random sampling within healthcare centres then used to select 631 participants (53.1% nurses, others : health officers, laboratory technicians, midwives, pharmacist)  In-depth interviews: 15 Key informants (nurses)  Practice observation: 15 health centres  Self-administered structured questionnaire  + key informants interviews + researcher observation of TBIPC practices | Quantitative: self-reported TBIC practice   1. Dependent variable = level of practice on TBIC 2. Independent variables included socio-economic factors, training, resources available, knowledge of IPC guidelines, use of PPE 3. high practice of natural ventilation 4. low use of PPE 5. low training of staff (lack of training– from quantitative studies as well) 6. high TB screening done because of current focus on this aspect of TB IPC practices 7. overall level of proper TB IPC practice was low (38%)   Qualitative:   - TBIPC policy not disseminated to staff - Administrative measures poorly implemented due to high workload and less priority on TBIPC - Environmental: insufficient space for isolation while awaiting transfer to MDR-TB facility, no engineers to design, maintain or install environmental controls - Selective training of health workers breeds the idea that only those who are trained are responsible for ensuring TBIC - Mostly comfortable with knowing own HIV (84.8%) and TB (89.2%) diagnosis - N95 only reserved for MDR cases - Individual: lack of motivation and negative attitudes, poor attitudes from patients - Predictors of adherence: department of work   Conclusion: TBIPC of healthcare workers was determined by 1) working area/department 2) knowledge status of TBIPC plan and presence of national TBIPC guideline in their health centers | - Despite a TBIPC officer appointed, there was no TBIC policy/plan or monitoring in place and it was not announced to all health workers – due to a lack of emphasis on the programme? - NGOs providing TBIPC training in collaboration with the government may have led to lower coverage - Tuberculosis infection control practices of health care workers was determined by their working area/department, knowledge status of TBIC plan and presence of national TBIC guide line in their health centers. |
| Chapman  Qualitative Health Research  2017  Dominican Republic | 1) To find out how clinicians who are familiar with TB management and guidelines use their knowledge as foundation when applying scientific concepts to the clinical setting.  2) To understand why “gaps” exist in practice.  3) Describe the decision-making process by which Healthcare workers use preventative strategies to reduce nosocomial TB transmission in their clinical practice  **Qualitative** | 40 Healthcare workers : 24 physicians, 16 nurses (only full time staff with minimum 1 year experiences)  Tertiary level health institution  Using grounded theory approach  Semi-structured interviews + FGDs of same sex and occupation, starting with discussion of 5 identified barriers, followed by recommendations to improve compliance and TBIPC measures | 1. Feeling powerless: unable to apply clinical knowledge to practice due to scarce resource, limited voice and inaction from health authorities 2. Intrinsic factors  - Two schools of thought: Moral obligation to promote adherence to TBIPC practices VS disregarding the role of infection control practices due to desire to help patients, forgetting about their own health and safety - Their perception of the risk diminishes with time  1. Extrinsic factors  - administrative policies: focus on training physicians, not providing care to patients and health providers - no incentive for continued education - Poor communication between the TB programme and their clinical department - Prioritisation of maternal-infant health indicators over TB incidence rates, although both are Sustainable Development Goals - Overburdened with clinical responsibilities - Institutional infrastructure - Families do not understand risks and need for Healthcare workers to obtain personal protective equipment - Healthcare workers question the costs of the masks and why health institutions are unable to purchase them - Aware that patients are unable to protect themselves | - knowledge−action gap influenced primarily by Healthcare workers’ feelings of powerlessness to prevent nosocomial M. tuberculosis transmission in their clinical practice - Although DR Ministry of Health has regularly updated and widely disseminated guidelines to improve hospital and community TB control, supervision of the application has not been prioritized - Healthcare workers knowledge and perceptions about TB infection and disease, however, limited their ability to protect themselves and their colleagues  1. Belief in “auto-vaccination” from habitual occupational exposure 🡪 feelings on invincibility 2. strong sense of vocation to maximize service delivery to patients, regardless of whether they were able to adhere to infection control measures 3. scientific and skill-based expertise do not equal experience or duration of employment  - Healthcare workers expressed dissatisfaction and disillusion that institutional health leaders, and stakeholders failed to identify and prioritize clear deficiencies in health care service delivery 🡪 feel devalued and unimportant 🡪 feel they have an insignificant role to play in TBIPC - their decision-making process to adhere to recommended infection control practices is influenced by their understanding of the clinical risk - Risk-benefit ratio may relate to the knowledge action gap - Recommendations  1. Provide opportunities in the workplace to pursue professional development with institutional support 2. Psychological motivation to maximize self-confidence 3. Building relationships to promote compassion and empathetic care 🡪 increasing confidence to apply leadership skills 4. The above 3 recommendations address to intrinsic level factors: the extrinsic or systems-level limitations within health institutions persist and should be promptly addressed |
| Chapman  MEDICC Review  2017  Dominican Republic | To identify perceived barriers to adherence to TBIPC measures among Healthcare workers in the Dominican Republic  **Qualitative** | Purposive sample of 7 physicians in internal or emergency medicine + 2 nurses all involved in TBIPC  2 tertiary level hospitals  Semi-structured individual interviews of nine questions conducted in Spanish, guided by socioecological framework | 5 barriers identified (Individual-level + institutional-level)   1. Individual-level 2. Sense of invincibility: due to BCG vaccine and clinical exposure OR fatalistic 3. Personal beliefs: empathy and building patient rapport take priority over wearing PPE, Spiritual faith as a protective factor 4. Institutional- level 5. Lower provider-to-patient ratio 6. Space limitation (infrastructure) 7. Limited availability of respiratory protection   Ineffective DOTS programme makes healthcare workers erroneously believe that patients are not infective + no regular screening for healthcare workers | - Healthcare workers may decide to relinquish personal protection in order to maximize perceived communication and compassionate care to patients while reducing associated stigma - There was evidence of a knowledge action gap, possible factors that expand the gap include: time-constrained or short-staffed health settings, healthcare workers’ limited knowledge or erroneous perceptions, lack of access to clinical research, political ideologies that conflict with scientific evidence, and lack of financial resources |
| Emerson  International Journal of Tuberculosis and Lung Disease  2016  Zambia and Botswana | To develop a TBIPC training and implementation of TBIPC activities in facilities implementing the package  Mixed method: **training course with pre and post-tests + key informant interviews** to identify implantation needs – we extracted the Qualitative section (in-depth interview)  *only a small section (the in-depth interview) was relevant to out aim | Zambia: 43 Healthcare workers from 8 facilities from 4 provinces  Botswana: 50 Healthcare workers from 10 Healthcare facilities from 5 districts  HCFs were some of the largest HIV clinics in the countries  Chosen by MoH leadership for their geographic representativeness and burden of disease  In depth interviews:   - 18 from Zambia - 51 from Botswana | Challenges   1. Human resources (attitudes and challenges of disseminating information): lack of Ic officer, or Ic officers with other responsibilities 2. Education and sensitization of patients 3. Facility structure – insufficient space inside, especially during rainy seasons 4. Finance: tissues, fans, extractors, N-95 masks   Suggestions made by Healthcare workers   1. Training and mentorship to staff: sensitization, frequent reminders 2. Community mobilization: materials for patients and relatives, training community treatment supporters 3. Structural improvements and maintenance: maintenance personnel, environmental modifications   Resources: specifically, DVD players and TVs for educational purposes | - Fixing poorly designed facilities would be costly and administratively challenging - Most facilities were able to accomplish the majority of TBIPC objectives within existing facility budgets - Recommendation: it may be helpful to develop a cadre of TB IC practitioners who can integrate broad-based TB IC issues and support the growth of in-country IC practitioner networks |
| Brouwer  PLos One  2014  Mozambique | To address the following questions   1. How do Healthcare workers perceive the occupational TB infection risk 2. What TBIPC measures do Healthcare workers report using to prevent TB transmission 3. What challenges do Healthcare workers report when using such measures   **Qualitative** | All facilities from 3 provinces represented (except provincial hospitals because they were thought too different from urban facilities)  86 Healthcare workers : axillary workers, doctors, clinical officers, nurses, TB programme staff purposively sampled to represent full range of healthcare  11 FGDs, min 5 participants per FGD conducted in Portuguese over 2 weeks (until saturation reached) | Risk perception:   - OPD nurses perceived themselves as being at a higher risk than TB nurses because they are less knowledgeable and hence less able to protect themselves - Concerned about infecting family members - Some feel the risk is higher due to frequency with which they encounter TB at work, other school of thought argues the risk is easily managed with precautionary measures - Feel the need for TBIPC in the community, not just in health facilities   TBIPC measures used by participants:   - All Healthcare workers felt health education was the most important measure - Except the nurses, all categories reported cough hygiene was most important - Except the TB staff, all categories reported that the use of respirators by healthcare workers were the most important measure (does not state what TB staff think are the most important measure) - The auxiliary staff and medical staff reported prioritization of patients with cough and providing good ventilation respectively (?in accordance with their roles?)   Challenges using IPC measures   - Healthcare system: infrastructure and availability of necessary materials or equipment and space - Regression of the practice of isolating TB patients due to it being “discriminatory” - Irregular supply of respirators 🡪 indifference 🡪 poor practice of respirator use - Difficulty in breaking old habits (e.g. incorrect use of respirators) - Barrier or distancing mechanism of respirators: - Patients: (1) everyday routines and socioeconomic factors - culturally difficult to stop having meals with their families (as in South African study) (2) behavior: using coughing to jump the queue   TBIPC training   - Limited resources therefore prioritization: training only TB staff, none for auxiliary staff | - Barriers are healthcare system related such as lack of clear guidelines and insufficient material and equipment - Also influenced by Healthcare workers and patient behaviour - There was substantial agreement between the different categories of Healthcare workers . Given the different roles and responsibilities of the Healthcare workers, the authors had anticipated more variety in what Healthcare workers reported on the challenges of TBIPC - programs need to concentrate more on improving Healthcare workers’ motivation and offer support of colleagues and supervisors - Behavior is not always the consequence of perfect logic. Social, emotional and environmental factors are all also relevant. - Healthcare workers are probably more likely to follow guidelines if they feel ownership of these guidelines 🡪 need involvement to create ownership |
| Buregyeya  BMC infectious diseases  2013  Uganda | To assess the implementation of TBIPC in facilities in Mukono and Wakiso disricts, whether facility characteristics were associated with implementation of TBIPC and identify barriers to implantation  **Qualitative + Observational** | Participants selected to represent staff from each category: doctors, clinical officers, nursing, midwifery, nursing aid, laboratory and radiographers (in facilities without all cadres represented, simple random sampling used)  51 facilities in 2 districts surrounding Kampala (semi-urban, predominantly rural): 543 participants out of 551 calculated sample size  Both hospitals and health centres  assess TBIPC measures implemented  2) Researcher observation of participant TBIPC practices    3) facility record checking  4) 8 FGDs (58 participants in total with 6-8 per group)   - 1 FGD males healthcare workers - 7 FGD female healthcare workers | Overall findings regarding TBIPC measures   - Managerial measures/facility level: Only 31% of facilities had a TBIPC plan (district dependent), those with plans also are more likely to provide employee training (general organization? Quality of Management?) - Administrative: 90% do not screen patients for cough neither are coughing patients given priority - Environmental: 44% did not have adequate ventilation in waiting areas - Personal Protective: No N95 masks in any of the 50 facilities   Barriers   - Structural: lack of space, tents provided for isolation. Tents broke. - Lack of Human Resources: understaffing 🡪 overstretched staff 🡪 screening becomes an additional burden - Stigma surrounding TB: hesitant to isolate suspected cases of TB prior to confirmed diagnosis - Managerial support: lacking funds for purchasing masks and implementing structural improvements 🡪 unable to separate HIV and TB patients - Negative attitude towards TB among Healthcare workers: Perceive no risk as they have been working in that environment for a long time and see no need for change/introduction of TBIPC measures - Lack of adherence among patients | - Administrative control measures are most important yet have not been implemented - **Huge discrepancy between reported and observed** (e.g. 43% and 55% reporting screening and separating TB suspects, but only 10% were observed implementing both) – shows they are aware of what to do, but fail to appreciate the importance of it, likely due to poor staffing levels - need for multi-pronged interventions in order to cause lasting behaviour change - Mukono district was doing better than Wakiso: difference may be attributed to the TB community annotation project 🡪 more comprehensive support in addition to training, and the more inclusive training |
| Turusbekova  Public Health  2016  Romania | To describe the implementation of a TBIPC project (to provide tailor-made technical assistance to TBIPC facilities and support them in developing their own functional TBIPC plans) aimed at helping practices and discuss its successes and challenges  **Interventional** (nation-wide project): Cross-sectional + qualitative components | Cross-sectional questionnaire (completed by 18/42 participants)  Collection of information on broader challenges done in an unstructured manner at training session with the 42 participants  Participants working at national level and TB facility level  Facility types not specified  Surveys filled in by participants + verbal feedback during training sessions | - Main challenges: (1) lack of authority of TBIC responsible person to implement the measures (2) lack of training among facility epidemiologists - Poor system for reporting TB among healthcare workers: underdeveloped or non-existant in some areas - Poor facility infrastructure – need to improve the use of natural ventilation: Mechanical ventilation even when installed was not maintained - Challenges in selecting and procuring respirators and use of surgical masks by patients - Lack of space and human resources make triaging difficult | - Making IC plans site specific to facility’s needs is the first and most important step to ensure adoption of measures by healthcare workers - Having a TBIPC plan does not automatically ensure a sense of local ownership among staff. This needs to be cultivated. This was done using developing the framework on principles of teamwork, co-design and co-production - No funding available for follow-up activities and supportive supervision - Need to sensitize key specialists at a national and subnational level by involving them in meetings on training |
| Woith  Internatinoal Journal of Tuberculosis and Lung Disease  2013  Russia | Identify barriers and motivators in the use of IC measures among Russian - healthcare workers  **Qualitative** | Convenience sample  3 TB hospitals + 2 TB outpatient clinics  96 healthcare workers (85% having worked in TB care facilities for more than 10 years)  Physicians, nurses, lab staff, support staff  15 homogenous FGDs (2 – 10 participants per group) assessed on:  (1) Knowledge of infectiousness and transmission of TB  (2) IPC methods used  (3) Barriers and motivators to TBIPC | Barriers   - K: Although physicians and nurses were most knowledgeable about TB and TB IC, there was generally poor knowledge of TB transmission among staff. Some think TB is spread by close contact with an infected person’s personal belongings, blood borne. Belief that a full stomach reduces susceptibility and exposure to cold increased susceptibility, believing lab coats and protective shoe coverings were protective - A: Negative attitudes about respirators - discomfort is most cited reason for non-adherence (sliding of mask, difficult to wear with rhinitis, perspiration and unattractive), interferes with sight (fogging of glasses, hinders ability to use microscope, interferes with talking to patients. - A: Initial fear of working with TB replaced by complacency when workers realize they are not going to get TB - P: Would rather wear surgical masks   Motivators   - K: continued education, maintaining a high standard of knowledge - A: main concern was infecting loved ones, loss of benefit payments if non-adherent to TBIPC, culture of taking care of themselves knowing no one else will look out for them, reminding each other to wear respirators - P: supportive behavior by administrators (conducting checks) | - Group pressure may help foster adherence, and rewarding the group for adherence would build on traditional collectivist approaches where the good of the group motivates worker behavior - Behavior change is also affected by perceptions of how others think one should behave - Workplace culture is linked to human resource development, which is critical for success of IC plans. However, cultural beliefs are less tangible than other barriers and may be more difficult to change. |
| Scott  International Journal of tuberculosis and lung disease  2017  Tajikstan, Central Asian Republics region. | To assess participants’ knowledge retention, sharing of training information, and changes in IC activities at participants’ workplaces following TBIPC training courses conducted during the first year of operation of the TBIPC National Training Center  **Interventional + Qualitative** (informal) | 84 (of 89 eligible) participants from a 4 day TBIPC course  (Kazakhstan, Kyrgyzstan, Tajikistan, Uzbekistan)  Type of Healthcare facility: TB center, Public health center, TB hospital, Epidemiological center, Health department, Medical university Laboratory, Penitentiary medical facility National hospital  Post-training review/interview on challenges faced in trying to implement IPC practices in their workplace | - Most frequently mentioned barrier: budget and funding (n = 29, 35%) hindering the expansion of TBIC practices, followed by environmental challenges (n = 13, 14%) and lack of TBIPC knowledge (n = 13, 14%). - top three suggestions for improving TBIPC training included providing additional TBIPC training (n = 16, 30%), advanced skills and refresher training (n = 14, 26%), and expanding the type of staff invited to training courses (n = 6, 11%) | - 77% reported that no TBIPC changes were made in their work facility after discussing needed changes with facility leadership - Participants’ limited knowledge retention of TB IC principles and practices observed during the evaluation indicate the potential advantage of tailored training courses. While some regression in knowledge scores over time is expected, the average decrease of 35 percentage points may reflect a lack of information utility. |
| Kanjee  Journal of Infection and Public Health  2012  South Africa, KwaZulu-Natal, Umzinyathi district (Rural) | To determine if the Motivation Behavioural Model can characterize TB IPC adherence and inform IC enhancement  **Cross-sectional**  F/U from study below done in 2007 🡪 SEE NEXT STUDY BELOW | Voluntary participation: clinical staff members working day shifts during the study period in high-risk departments of the hospital  198 healthcare workers in 2 hospitals  Nurses (78.1%), physicians, student nurses, nursing assistants  Questionnaire: 45 minute survey based on information, motivation and behavioral skills model (IMB) | - Information: Good to moderate, scores were hospital dependent  1. 34.3% believed that surgical masks offer same protection as respirators 2. 54.0% thought that mechanical ventilation is always better than natural ventilation 3. Majority of the respondents (69%) did not know that healthcare workers with HIV can contract TB even though they practiced TB IC.  - Motivation: 29.4% not bothered about catching TB, 19.3% felt less susceptible to TB than their colleagues, 22.8% did not think TB IC was worthwhile, 44.6% found respirators uncomfortable, 30.4% felt it ruined their appearance, 27% felt that HIV positive healthcare workers would be severely stigmatised, 31.6% felt their colleagues would find out their HIV test results if they were to get tested at the facility. Numbers higher for TB, although 98.5% agreed that knowing your HIV status is important - Behavioural: most behavioural skills rated as easy – opening windows, acquiring respirators and opening windows. Personal HIV and TB diagnosis and dealing with stigma, or trusting managers to maintain HIV/TB status confidentiality all deemed relatively difficult | - Motivation and behavioral skills most associated with TB IC practice, NOT information - Perspectives and actions of co-workers can influence Healthcare worker behavior: fear of ostracisation - Supervisors are essential as (1) role models (2) rewarding Healthcare worker efforts (3) providing effective performance feedback - Information has a ceiling effect (or wrong information being assessed?) |
| Kanjee  Journal of Hospital infection  2011  South Africa | Baseline audit of KAP - information gathering on ICP measures  **Cross-sectional + practice-observation** | Convenience sample  57 healthcare workers, including all categories of nurses and community healthcare workers (40.4% from TB wards)  1 resource- limited rural district hospital  Baseline audit of KAP with 100-item Self-administered questionnaire + unannounced direct observations conducted by researchers (10 hours) | Discrepancy between understanding importance of TBIPC and feeling comfortable carrying out the TBIPC measure, mostly markedly with knowing their own HIV/TB diagnosis (HIV>TB)  Discrepancy between reported and observed practices  Barriers:   - Close to half of the respondents (49.1%) felt that the hospital administration did not care for them and was not proactive in preventing TB infection in staff - As a result of lack of formal policy, 37.5% were less likely to follow TB IC recommendations - All respondents agreed that it was very important to prevent the spread of TB at the hospital. - 13.2% reported doing cough hygiene often - Patients complain when windows and doors open - Fear of stigma with their own TB/HIV diagnosis: not wanting to disclose status to supervisors - Lack of trust in doctors maintaining confidentiality regarding TB/HIV status - Insufficient N95> discomfort of N95 | Multipronged interventions are most consistent with lasting behavior change  No analysis carried out on the KAP scores, the relationship between knowledge and practice or attitude and practice |
| Engelbrecht  BMC Infectious Diseases  2016  South Africa, Free State province | To determine the factors associated with healthcare workers’ good TB infection control practices in PHC facilities  **Cross-sectional + practice-observation** | Convenience sampling: questionnaire distributed to all categories of nurses and facility-based community healthcare workers (CHCWs)  236 participants = 34 CHCWs + 202 nurses (about half of each population captured)  41 PHC facilities  KAP self-administered questionnaire aimed at facility, admin, environmental and personal protection + facility TBIPC practice observations  (In the 12 months prior to the study, ¾ of the respondents had received HIV and TB related training, including infection control related training) | - Pearson’s chi-square test revealed association between knowledge and TB practices - Knowledge: good to average, however there were certain areas where there was poor knowledge: Less than a quarter of the respondents knew the following:  1. that HIV positive healthcare workers could still become ill with TB even if they practice IPC 2. wearing surgical mask did not protect healthcare workers from acquiring TB  - Attitudes: 80.4% positive attitudes, but 84.6% feared acquiring TB at work, 3/10 felt clinic unconcerned for their health and safety BUT 87% screened at work - Practices: over reported level of environmental IPC measures (discrepancy between self-reported and observed data) - Every unit increase in attitude = 1.090 times increase in good practice (CI 1.016 – 1.169) - High levels of knowledge (>80% right answers) = 4.029 times more likely to have good practice - Environmental infection control better implemented than administrative or personal protective measures | - Discrepancy between self-reported and observed TBIPC practices - lack of attention to the implementation of administrative control measures is a serious concern given that these are the first line of defense in healthcare facilities. These measures are simple and not costly to implement - attitudes and good levels of infection control knowledge were statistically significant predictors of good practices - Training was not found to be a good predictor of good practice 🡪 calls into question type and quality of training, that perhaps training is too theoretical rather than skills-based - Support and mentorship should go along with training |
| Engelbrecht  African Journal of Primary Healthcare & Family Medicine  2018  South Africa | To describe the state of implementation of TB infection control measures in a high-burden metro in South Africa  *(Hence only a small section of this paper was relevant given that the aim differs greatly from ours)*  **Cross-sectional** | TB nurses purposively selected due to their knowledge on the state of TBIPC practices in PHC facilities  41 TB nurses  41 PHC facilities  Researcher administered questionnaire | - Main focus was on evaluating implementation which is not relevant to our systematic review   Small paragraph stating barriers to effective implementation of TBIPC practices in primary health care facilities.   1. Lack of equipment: disposable respirators and disposal containers 2. Poor facility layout causing lack of space for separate waiting areas – coughing patients walk through the entire facility to reach the TB room 3. Poor cough etiquette of patients 4. Poor staff attitudes towards IPC | 1. In order for PHC Healthcare workers to effectively implement TB infection control, all healthcare system components are required to function well: governance and stewardship, financing, infrastructure, procurement and supply chain management, human resources, health information systems, service delivery and finally supervision. 2. This paper analysed the barriers using the managerial, administrative, environmental, PPE model, and emphasized the importance of training for all four categories to allow implementation at each level 3. Poor systematic collection of data at a national level |
| Westhuizen  International Journal of Infection Control  2015  South Africa, Stellenbosch University | To measure the impact of novel educational intervention on students’ awareness of TB infection risk and knowledge of TB IC  **Interventional + Cross-sectional (KAP)**: group educational sessions on TB | Convenience sample  326 participants = 259 medical students + 67physiotherapy students (2^nd^ – 4^th^ year of training in Capetown)  Students at Stellenbosch University, South Africa  Self-administered KAP questionnaire based on health belief model (perceived susceptibility, perceived severity, perceived benefits, perceived barriers, cues to action and self-efficacy) | - Barriers: authority of seniors “overruled by superior”, (73%) duty of care to patients, feelings of powerlessness (fear of it affecting exam scores (66%) - Senior students have better knowledge scores, (from 58% - 78%) suggesting that a degree of education regarding respirator use was taking place in clinical environment - Attitude change post-intervention: using occupational TB survivors - Healthcare workers were perceived by students as poor role models | - Educational intervention was at least partly successful in increasing TBIPC knowledge and changing attitudes - Incorrect practices among senior colleagues appeared to adversely affect students’ perceptions of the importance of TBIPC - improved knowledge and awareness of TBIPC measures can be achieved using novel education methods |
| Temesgen  BMC Health Services Research  2014  Northwest Ethiopia | To assess TBIPC knowledge and practices among health professionals working in hospitals in the Amhara region of Northwest Ethiopia  **Cross-sectional** | 4 hospitals selected based on flow of TB patients.  326 (of 498 healthcare workers in hospitals)  Physicians, health officers, nurses, lab technicians, pharmacists, radiographers, physiotherapists, environmental health workers  Pre-tested structured questionnaire related to knowledge and practice of TBIPC | - Only 18.8% of the respondents were trained on TBIPC - Generally good knowledge apart from respirator and fan (ventilator) use - Training was the strongest determination of knowledge - Knowledge was a strong predictor of good TBIPC practice AOR 10.667, 95%CI (5.769,19.721 - Job location and age had no effect on knowledge | - Good TBIPC knowledge and practices and good knowledge about need for TBIC committee - TB IC training = good knowledge, good knowledge = good TBIPC practice - Yet training on TBIC on its own does not appear to have any positive influence on TBIC practice, likely due to emphasis on theoretical rather than skill-based component |
| Mirtskhulava  Infection Control Hospital Epidemiology  2015  Georgia | To provide baseline data on knowledge, beliefs and behaviors related to TBIPC, which will later be used to develop and implement TBIPC interventions/programs at Georgian HCFs  **Cross-sectional** | Convenience sampling  Healthcare workers in the National TB Programme or PHC  Conducted prior to TB education  240 Healthcare workers , anyone working in a healthcare facility  55 question survey developed based on the Health Belief Model covering  (1) TB knowledge  (2) Health Related behaviors  (3) willingness to engage in health-related behavior change  (4) perceived susceptibility to and severity of LTBI and TB disease | - Only 43% of Healthcare workers knew the risk of LTBI progression to TB - Only 30% able to correctly identify high risk TB groups - Only 36% of 78% of Healthcare workers from the National TB programme reported frequent use of respirators even when around patients with risk of TB or who had active TB - More knowledge and awareness of LTBI and TB disease (risk of progression, rationale behind treatment and diagnostics) = more likely to think screening is important and more likely to worry about acquiring TB - Predictors of TBIC related behavior: Healthcare workers more likely to refuse treatment for LTBI if they worked in TB facilities 🡪 perceived high personal risk of TB reinfection - Those who perceived LTBI as a more serious threat were more willing to be treated for LTBI - Availability of respirators was the only significant predictor of use - Adherence more likely if perceived susceptibility to TB infection and net benefit of intervention is higher | - physicians compared to nurses were found to have greater knowledge related to TB and TBIPC measures - Healthcare workers knowledgeable about TB and TBIPC measures were more likely to perceive their susceptibility to TB infection, severity of TB disease, and TBIPC intervention benefits and barriers - As TB services are currently being integrated in PHCs as part of the ongoing health system reforms in Georgia, all nurses (both working with TB patients and non-TB healthcare facility nurses) need training on TBIPC - Researchers and healthcare facility administrators should pursue the application of behavioral science methods to strengthen TBIPC measures implementation process |
| Mtech et al  American Journal of Infection Control  2015  Lesotho | To assess the level of adherence to TBIPC and the factors associated with nonadherence amongst nurses  **Cross-sectional** | Purposively sampled nurses working in TB wards and outpatient departments  55 nurses (out of 120-member nursing staff)  2 referral district hospitals (Motebang and Mafeteng Hospitals) each with a TB ward  Semi-structured questionnaire | - 43.6% non-adherent to guidelines - 22% reported inaccessibility of guidelines which were in the custody or particular nurses - Fear was a significant variable in determining adherence to guidelines  1. Lack of equipment (2 main barriers) 2. Inadequate staff (2 main barriers) 3. Lack of clarity of guidelines 🡪 implies gaps in training 4. Time constraints (related to staff shortage?)   Only 6% reported no issues with IC guidelines  Conflicting responses about frequency of IC meetings. 10% did not know of their existence | - Fear of occupational exposure could result in default adherence to IPC guidelines or could risk providing substandard care to patients - Insufficient nursing staff leads to incorrect equipment setup and nurse exhaustion, increases workload, compromising the quality of services - Infection control committee needs to fulfill their role: design and appropriate use of facility, and to conduct surveillance of TB amongst Healthcare workers - Insufficient patient education implies, lack of awareness of its importance in TB infection control |

**Abbreviations**

TBIPC Tuberculosis infection prevention and control

DOTS Directly observed treatment, short-course

FGD Focus group discussion

OH Occupational health

DR-TB Drug resistant tuberculosis

IMB Information-motivation-behavioural

OPD Outpatient department

KAP Knowledge, attitudes and practice

PHC Primary healthcare

LTBI Latent tuberculosis infection
