# Supplementary material for: Barriers and facilitators of tuberculosis infection prevention and control in low- and middle-income countries from the perspective of healthcare workers: A systematic review
Source: PLoS One. 2020 Oct 21;15(10):e0241039. doi: 10.1371/journal.pone.0241039 (PMC7577501; doi:10.1371/journal.pone.0241039)
Supplement: S2 Table — (DOCX) [file pone.0241039.s002.docx]

**S2 Table. Quality Assessment Tool for Quantitative Studies by the Effective Public Health Practice Project (EPHPP)**

| **Component Ratings** | Kanjee  2012 | Kanjee  2011 | Engelbrecht  2016 | Engelbrecht 2018 | Westhuizen  2015 | Temesgen  2011 | Mirtskhulava  2012 | Mtech  2015 |
| --- | --- | --- | --- | --- | --- | --- | --- | --- |
| **A) SELECTION BIAS**  (Q1) Are the individuals selected to participate in the study likely to be representative of the target population?  1 Very likely 2 Somewhat likely 3 Not likely 4 Can’t tell | 1  (84% female, 78% nurses) | 2  (75% female, 44% nurses, | 1  (study on nurses only) | 1  (TB nurses only) | 1  (Study on students only) | 1 | 1  (Majority of staff within the HCF were female) | 3  (excluding nurses who worked less than 1 month) |
| (Q2) What percentage of selected individuals agreed to participate?  1 80 - 100% agreement 2 60 – 79% agreement 3 less than 60% agreement  4 Not applicable 5 Can’t tell | 1 | 5 | 3  (49.9%) | 1 | 5  (convenience sample) | 1  (96%) | 1  (81%) | 3  (45.8%) |
| **SELECTION BIAS**  1 Strong 2 Moderate 3 Weak | **1** | **3** | **3** | **1** | **2** | **1** | **1** | **3** |
| **B) STUDY DESIGN** | Cross-sectional  (IMB) | Cross-sectional  (KAP) | Cross-sectional  (KAP) | Cross-sectional and observational  (structured interview) | Cohort study  (KAP) | Cross-sectional  (KAP) | Cross-sectional  (KAP) | Cross-sectional |
| **STUDY DESIGN**  1 Strong 2 Moderate 3 Weak | **3** | **3** | **3** | **2** | **2** | **3** | **3** | **3** |
| **C) CONFOUNDERS**  (Q1) Were there important differences between groups prior to the intervention? (Department, Job title, Career length etc.)  1 Yes 2 No 3 Can’t tell | 1 | 1 | 1 | 2  (different levels of education) | 1 | 1 | 1 | 1 |
| (Q2) If yes, indicate the percentage of relevant confounders that were controlled (either in the design (e.g. stratification, matching) or analysis)?  1 80 – 100% (most) 2 60 – 79% (some) 3 Less than 60% (few or none)  4 Can’t Tell | 4 | 4 | 1 | 1 | 1 | 1 | 1 | 1 |
| **CONFOUNDERS**  1 Strong 2 Moderate 3 Weak | **3** | **3** | **1** | **1** | **1** | **1** | **1** | **1** |
| **E) DATA COLLECTION METHODS**  (Q1) Were data collection tools shown to be valid? (valid if it measures what it intends to measure)  1 Yes 2 No 3 Can’t tell | 1 | 1 | 1 | 1 | 3 | 1 | 1 | 3 |
| (Q2) Were data collection tools shown to be reliable? (reliable if the same result is obtained from using the method on repeated occasions)  1 Yes 2 No 3 Can’t tell | 3 | 3 | 1 | 1 | 3 | 1 | 1 | 3 |
| **DATA COLLECTION METHOD**  1 Strong 2 Moderate 3 Weak | **2** | **2** | **1** | **1** | **3** | **1** | **1** | **3** |
| **F) WITHDRAWALS AND DROP-OUTS**  (Q1) Were withdrawals and drop-outs reported in terms of numbers and/or reasons per group?  1 Yes 2 No 3 Can’t tell 4 Not Applicable (i.e. one time surveys or interviews) | 4 | 4 | 4 | 4 | 2 | 4 | 4 | 4 |
| (Q2) Indicate the percentage of participants completing the study. (If the percentage differs by groups, record the lowest).  1 80 -100% 2 60 - 79% 3 less than 60% 4 Can’t tell  5 Not Applicable (i.e. Retrospective case-control) | 1 | 5 | 5 | 1 | 1 | 5 | 5 | 5 |
| **WITHDRAWALS AND DROP-OUTS**  1 Strong 2 Moderate 3 Weak | **NA** | **NA** | **NA** | **NA** | **NA** | **NA** | **NA** | **NA** |
| **H) ANALYSES**  (Q1) Indicate the unit of allocation  community organization/institution practice/office individual | Individual | Individual | Individual | Individual | Individual | Individual | Individual | Individual |
| (Q2) Indicate the unit of analysis  community organization/institution practice/office individual | Individual | Individual | Individual | Individual | Individual | Individual | Individual | Individual |
| (Q3) Are the statistical methods appropriate for the study design?  1 Yes 2 No 3 Can’t tell | 1  Correlations  between IMB variables and self-reported personal  TBIPC practices were assessed using Spearman correlations  and Wilcoxon scores. Each self-reported  practice was regressed in a path analytic model  against a pre-planned set of IMB items that was  hypothesized to be conceptually related. These  structural equation models were examined for magnitude  and direction of paths. | 2  No data were described using frequency counts and percentages | 1  Binomial logistic regression analysis used to determine factors associated with good TBIPC practice | 2  Data were described using frequency counts and percentages for categorical variables and means and  standard deviations for continuous variables. Composite  scores were calculated for the four levels of infection control | 1  Two-way mixed model repeated measures ANOVA  was conducted to compare ordinal measurements  between two time points | 1  Using SPSS 15,  frequencies, percentages,  and means calculated for TBIPC  knowledge, and practice. Percentage compliance  was calculated. The relationships of independent/  predictor variables  with dependent variables  were calculated  through cross tabulation and a summary table was  generated. Univariate binary logistic analysis and multivariate  logistic regression analysis used to  determine the relationship between outcome variables  and a range of factors | 1  Using SPSS 19, calculated frequency distributions; if <10% of participants responded to a question item, that item was excluded from further analysis. Five-level variables measuring HCWs beliefs about TBIPC measures were reduced to three-level variables for multivariate analysis. Using binomial logistic regression to estimate the association between HCW demographic characteristics and knowledge of TB; ordinal (when proportional odds assumption was met) or multinomial logistic regression were used to estimate the association between HCW’s beliefs and their behaviors. Collinearity was assessed for multivariable models, variables with significant collinearity were removed. Mann-Whitney U-test to compare the median scores of HCWs’ beliefs among two independent groups | 1  Logistic regression analysis used to account for lack of adherence |
| (Q4) Is the analysis performed by intervention allocation status (i.e. intention to treat) rather than the actual intervention received?  1 Yes 2 No 3 Can’t tell | NA | NA | NA | NA | 1 | NA | NA | NA |
| **GLOBAL RATING FOR THIS PAPER**  1 Strong 2 Moderate 3 Weak | 3 | 3 | 3 | 1 | 2 | 2 | 2 | 3 |

**Blinding and Interventional Analysis were not used due to the nature of the studies.**

**Abbreviations**

TB Tuberculosis

IMB information, motivation, and behavioral skills

KAP Knowledge, attitude and practice

NA Not applicable
